# Seizure frequency, APOE ε4, and cognitive function in older people with epilepsy

**DOI:** 10.1186/s42494-025-00213-7

**Published:** 2025-05-23

**Authors:** Yiling Chen, Zhenxu Xiao, Xiaowen Zhou, Saineng Ding, Luxin Jiang, Qianhua Zhao, Ding Ding, Jianhong Wang, Guoxing Zhu

**Affiliations:** 1 Department of Neurology, Huashan Hospital, Fudan University, 12 Wulumuqi Middle Road, Shanghai, 200040 China; 2 National Clinical Research Center for Aging and Medicine, Huashan Hospital, Fudan University, 12 Wulumuqi Middle Road, Shanghai, 200040 China; 3 National Center for Neurological Disorders, Huashan Hospital, Fudan University, 12 Wulumuqi Middle Road, Shanghai, 200040 China; 4 Institute of Neurology, Huashan Hospital, Fudan University, 12 Wulumuqi Middle Road, Shanghai, 200040 China; 5 Department of Neurosurgery, Huashan Hospital, Fudan University, 12 Wulumuqi Middle Road, Shanghai, 200040 China

**Keywords:** Epilepsy, *APOE*, Older adults, Seizure frequency, Cognitive impairment

## Abstract

**Background:**

Cognitive impairment represents a major comorbidity among older adults with epilepsy. This study aimed to explore the association between the apolipoprotein E (*APOE*) ε4 allele and cognitive function in older people with epilepsy.

**Methods:**

People with epilepsy aged ≥ 50 years were enrolled at an outpatient clinic of epilepsy from November 2019 to July 2024. Blood samples were collected for APOE genotyping. Participants were categorized into two groups based on the presence of the *APOE ε4* allele: *APOE ε4* (+/-). Cognitive function was assessed using a battery with neuropsychological tests. Based on Mini-Mental State Examination (MMSE) scores, participants were defined as unimpaired cognition (UC) (MMSE ≥ 27) and cognitive impairment (CI) (MMSE < 27). Seizure frequency was categorized into low (≤ 3/year) and high (> 3/year) groups. Multivariate logistic regression analysis and general linear models were employed to identify factors associated with cognitive function.

**Results:**

Among 110 participants, 51 (46.4%) were defined as CI. Compared with UC group, the CI group was older (65.1 ± 7.6 vs 60.8 ± 6.8 years, *P* = 0.002), with lower educational level (9.0 [7.0, 11.0] vs 12.0 [9.0, 13.0] years, *P* < 0.001), and higher seizure frequency (12.0 [1.0, 24.0] vs 1.0 [0.0, 12.0] times/year, *P* = 0.005). High seizure frequency (OR = 3.94, 95% CI [1.34, 11.61], *P* = 0.013) and more *APOE ε4* alleles (OR = 3.28, 95% CI [1.09, 9.83], *P* = 0.034) were risk factors for CI. An interactive effect between the number of *APOE ε4* alleles and seizure frequency was observed (*P* = 0.002). Compared to participants with *APOE ε4* (-) and low seizure frequency, those with *APOE ε4* (-) and high seizure frequency showed a threefold risk of CI (OR = 3.34, 95% CI [0.99, 11.25], *P* = 0.051), while those with *APOE ε4* (+) and high frequency demonstrated the highest risk of CI (OR = 10.53, 95% CI [1.75, 63.47], *P* = 0.010).

**Conclusions:**

The synergistic effect of *APOE ε4* allele and seizure frequency on cognitive function suggested their importance in clinical assessments and therapeutic approaches in managing older people with epilepsy.

## Background

Epilepsy is one of the common neurological disorders, with a prevalence of approximately 6 per 1000 persons and an incidence of 68 per 100,000 person-years [1]. The incidence of epilepsy increases steadily after 50 years of age, and is up to approximately twice that of younger adults [2–4].

Epilepsy and its comorbidities pose a tremendous burden in older people with epilepsy and the society. Epilepsy is considered to have a potential bidirectional association with Alzheimer’s disease (AD), suggesting that the cognitive impairment in people with epilepsy may have similar pathological changes with AD [5, 6]. This notion implies a need for further research to elucidate the mechanisms underlying these conditions and to explore potential therapeutic targets that could address both cognitive decline and epilepsy.

Apolipoprotein E (*APOE*) gene, considered as a genetic factor of AD, is also associated with epilepsy. Compared with other genotypes, *APOE ε4/ε4* carriers have the smallest neurons, the lowest acute phase responses, the highest markers of stress, and lower level of intraneuronal APOE protein. This diminished APOE protein can promote aggregation of amyloid [5], and further impair neuronal repair and increase the seizure susceptibility [5, 7]. The proportion of *APOE ε4* allele is higher in people with epilepsy, especially those with temporal lobe epilepsy (TLE), compared to normal people [8].

As a clinical characteristic of epilepsy, seizure frequency has also been shown to increase the risk of cognitive impairment and dementia, especially in individuals with no prior cognitive problems or mild cognitive impairment [9]. In this study, we intended to verify the hypothesis that cognitive function may be impacted by *APOE ε4* allele and seizure frequency in older people with epilepsy.

## Methods

### Study participants

**Fig. 1:**
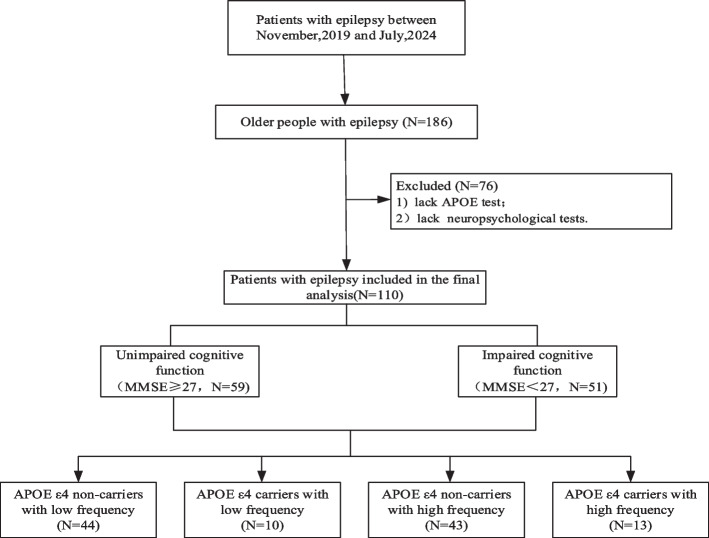
Flowchart of participants recruitment for the study. Abbreviation: APOE ε4: Apolipoprotein E ε4; MMSE: Mini-Mental Status Examination

We enrolled older people with epilepsy from the outpatient epilepsy clinic at Huashan Hospital, Shanghai, between November 2019 and July 2024. Inclusion criteria were as follows: 1) age ≥ 50 years; 2) met the 2017 International League Against Epilepsy diagnostic criteria for epilepsy [10]; and 3) agreed to participate in this study. Participants were excluded if they: 1) were diagnosed with severe brain trauma or cerebrovascular disease; 2) were diagnosed with a neurological disorder that could affect cognitive function, such as AD or Parkinson’s disease [11], or had a history of severe mental disorders. In the current study, we also excluded participants who: 1) had significant hearing or visual impairments that would preclude participation in cognitive assessments; 2) did not provide blood samples for *APOE* genotyping test. The procedure of recruitment is shown in Fig. 1.

### Demographic data and clinical characteristics

Demographic data was collected through a questionnaire administered by trained health professionals during in-person visits, including age, gender, and education year.

Clinical characteristics were collected through inquiry and questionnaire, including age at onset, disease duration, using ASMs or not, seizure frequency, and seizure type. The frequency of seizures in the previous year was retrospectively reviewed. Seizure freedom for more than one year was used as the criterion for well-controlled epilepsy. In this study, participants were categorized into two groups based on the median of seizure frequency in the previous one year: low seizure frequency (≤ 3 seizures per year) and high seizure frequency (> 3 seizures per year).

We collected detailed medical history of hypertension, diabetes, and dyslipidemia through a combination of medical record reviews and health questionnaires.

### APOE genotype assessment

DNA was extracted from blood, and *APOE* genotyping was conducted by the TaqMan single-nucleotide polymorphism method [12]. Participants with at least one *APOE ε4* allele were categorized as *APOE ε4* (+), whereas those devoid of any *APOE ε4* allele were classified as *APOE ε4* (-).

### Neuropsychological testing

Cognitive function was assessed using the Huashan version battery of the neuropsychological tests, which has been validated through rigorous reliability and validity testing in Chinese population [13].

a The Chinese version of the Mini-Mental Status Examination (MMSE) was used to assess global cognitive function [14]. Participants were further divided into two groups according to the median score of MMSE: unimpaired cognition (UC) (MMSE score ≥ 27) and cognitive impairment (CI) (MMSE score < 27).
b The Auditory Verbal Learning Test (AVLT) or Huashan Object Memory Test (HOMT) was used to measure verbal memory. For participants with < 6 years of education, the HOMT was administered, whereas for those with ≥ 6 years of education, the AVLT was chosen for assessment. Participants were asked to recall 12 words over 5 trials. We used the results of the 5th trial as the long-term delayed recall to represent the memory function [15].
c Trail Making Tests A (TMT-A) and B (TMT-B) were adopted to evaluate the attention and executive function, respectively [13].The test consists of two parts: TMT-A, participants needed to connect all numbers in sequential order; TMT-B, participants were asked to connect numbers in sequential order with the alternation of square and circle (e.g., “1” in a square, “2” in a circle). The completion time (seconds) was recorded and transformed to speed (1/time).
d The Stick Test was used to evaluate the visuospatial function [13]. Participants were asked to copy the examiner’s model exactly with four wooden sticks. After that, participants were asked to construct the reverse pattern of the model.
e The Modified Common Objects Sorting Test (COST) was used to assess the language function [13]. Participants were asked to sort all 42 objects into 7 different groups, and provided the reason for the sorting.
  Scores of each domain-specific test were standardized to *z* scores by subtracting the mean and dividing by the standard deviation (SD).

### Statistical analysis

Normally distributed continuous data are presented as Mean ± SD, while skewed distribution continuous data are expressed as Median (IQR). Categorical variables are represented as *n* (%). Significance analysis for continuous data was conducted using student *t*-test or Mann-Whtiney U-test, and Pearson chi-square test was applied for analysis of categorical variables. Multivariate logistic regression model adjusted for confounders (age, sex, years of education, epilepsy duration, hypertension, diabetes, and dyslipidemia) was used to test the association between seizure frequency, APOE ε4, and cognitive function. General Linear Model was used to explore the synergistic effects of seizure frequency and APOE ε4 on cognitive function, adjusting for age, gender, years of education, disease duration, seizure type, hypertension, diabetes, and dyslipidemia. To evaluate the combined effect of APOE ε4 status and seizure frequency on cognitive impairment, we constructed a categorical variable “Group”, with four levels (*APOE ε4* [-] and low seizure frequency, *APOE ε4* [-] and high seizure frequency, *APOE ε4* [+] and low seizure frequency, and *APOE ε4* [+] and high seizure frequency) based on the interaction between these factors. This “Group” variable was then incorporated as a factor in a univariable and a multivariate logistic regression models adjusted for age, gender, educational level, duration of epilepsy, seizure type, hypertension, diabetes, and dyslipidemia, and estimate odds ratios (OR) for cognitive impairment across the other levels of the “Group” variable relative to the reference group with “*APOE ε4* (-) and low seizure frequency”.

SPSS 26.0 was used for data analysis. Two-sided *P* < 0.05 was considered statistically significant.

## Results

### Characteristics of study participants

This study included 110 older people with epilepsy (55 males, 55 females). The age of all participants ranged from 50 to 81 years (mean 62.8 ± 7.5 years). The median duration of epilepsy was 12.5 years (IQR 5.0, 32.0), with a median age at onset of 48.5 years (IQR 30.8, 61.0). The majority of participants (96.4%) had focal seizures, and the rest had generalized seizures. Six participants (5.5%) discontinued anti-seizure medication (ASM) during the course of the disease. Thirty-three participants (30.0%) were seizure-free for one year, whereas 56 participants (50.9%) had four or more seizures per year. There were 20 participants (18.2%) with one *APOE ε4* allele and 3 participants (2.7%) with *APOE ε4/ε4*. In terms of comorbidities, 30 participants (27.3%) had a history of hypertension, 7 (6.4%) had diabetes, and 38 (34.5%) had dyslipidemia. The median MMSE score was 27.0 (IQR 25.0, 28.0).

As shown in Table 1, compared with the UC group, CI participants were older (65.1 ± 7.6 vs 60.8 ± 6.8 years, *P* = 0.002), had a lower level of education (9.0 [7.0, 11.0] vs 12.0 [9.0, 13.0] years, *P* < 0.001), and had a higher seizure frequency (12.0 [1.0, 24.0] times/year vs 1.0 [0.0, 12.0] times/year, *P* = 0.005). The differences between the two groups in cognitive domains are mainly shown in global cognition (24.0 [21.0, 26.0] vs 28.0 [28.0, 29.0], *P* < 0.001), memory (-0.4 [-1.3, 0.1] vs 0.4 [-0.4, 1.3], *P* < 0.001), attention (-0.6 [-1.1, -0.1] vs 0.4 [-0.3, 1.2], *P* < 0.001), executive function (-0.5 [-0.4, 0.9], *P* < 0.001), visuospatial function (-0.4 [-0.7, 0.4] vs 0.34 [0.0, 1.0], *P* < 0.001), and language (-0.1 [-0.5, 0.4] vs 0.4 [0.1, 0.6], *P* < 0.001).

**Table 1:** Characteristics of the participants of unimpaired cognitive function and impaired cognitive function

	Unimpaired cognitive function (*n* = 59)	Impaired cognitive function (*n* = 51)	*P*-value
Demographic information			
Age, years, M ± SD	60.8 ± 6.8	65.1 ± 7.6	0.002
Gender			0.566
Men, *n*(%)	28 (47.5)	27 (52.9)	
Women, *n*(%)	31 (52.5)	24 (47.1)	
Education, years, median (IQR)	12.0 (9.0, 13.0)	9.0 (7.0, 11.0)	< 0.001
Clinical information			
Age at onset, years, median (IQR)	48.0 (29.0, 59.0)	49.0 (31.0, 65.0)	0.417
Duration, years, median (IQR)	11.0 (5.0, 30.0)	14.0 (5.0, 33.0)	0.688
Using ASMs, *n* (%)	56 (94.9)	48 (94.1)	> 0.999
Seizure frequency, times/year, median (IQR)	1.0 (0.0, 12.0)	12.0 (1.0, 24.0)	0.005
Seizure type			0.717
Focal onset, *n* (%)	56 (94.9)	50 (98.0)	
Generalized onset, *n* (%)	3 (5.1)	1 (2.0)	
Clinical health parameter			
Hypertension, *n* (%)	14 (23.7)	16 (31.4)	0.369
Diabetes, *n* (%)	3 (5.7)	4 (8.9)	0.822
Dyslipidemia, *n* (%)	25 (42.4)	13 (25.5)	0.063
*APOE* genotype			
Number of *APOE ε4*			0.057
0, *n* (%)	50 (84.7)	37 (72.5)	
1, *n* (%)	9 (15.3)	11 (21.6)	
2, *n* (%)	0 (0.0)	3 (5.9)	
*APOE ε4* allele			0.117
APOE ε4 (-), *n* (%)	50 (84.7)	37 (72.5)	
APOE ε4 (+), *n* (%)	9 (15.3)	14 (27.5)	
MMSE, median (IQR)	28.0 (28.0, 29.0)	24.0 (21.0, 26.0)	< 0.001
Domain-specific cognition^a^			
Memory, median (IQR)	0.4 (-0.4, 1.3)	-0.4 (-1.3, 0.1)	< 0.001
Attention, median (IQR)	0.4 (-0.3, 1.2)	-0.6 (-1.1, -0.1)	< 0.001
Executive function, median (IQR)	0.2 (-0.4, 0.9)	-0.5 (-1.2, -0.0)	< 0.001
Visuospatial function, median (IQR)	0.3 (0.0,1.0)	-0.4 (-0.7, 0.4)	< 0.001
Language, median (IQR)	0.4 (0.1, 0.6)	-0.1 (-0.5, 0.4)	< 0.001

*Abbreviations*: *M* Mean, *SD* Standard division, *IQR* Interquartile range, *ASM* Antiseizure medication, *APOE* ε4, Apolipoprotein E ε4, *MMSE* Mini-Mental Status Examination

^a^Domain specific tests were standardized to *z* scores

### Association between seizure frequency, APOEε4 and cognitive function

After adjusting for age, gender, years of education, disease duration, seizure type, hypertension, diabetes, and dyslipidemia, high seizure frequency (OR = 3.94, 95% CI [1.34, 11.61], *P* = 0.013) and the number of *APOE ε4* alleles (OR = 3.28, 95% CI [1.09, 9.83], *P* = 0.034) were found to be associated with CI, respectively.

### Additive effect of seizure frequency and *APOE ε4* on cognitive function

The General Linear Model showed an interaction between seizure frequency and the number of *APOE ε4* alleles (F = 6.709, *P* = 0.002) on MMSE score.

As shown in Fig. 1, based on seizure frequency and *APOE ε4* carrier status, we stratified participants to four distinct groups: *APOE ε4 *(-) and low frequency (*N* = 44), *APOE ε4* (-) and high frequency (*N* = 43), *APOE ε4* (+) and low frequency (*N* = 10), and *APOE ε4* (+) and high frequency (*N* = 13).

**Fig. 2:**
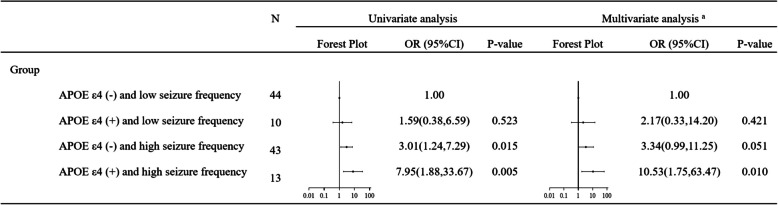
Interaction between *APOE ε4* allele status and seizure frequency in risk of cognitive impairment. Abbreviations: *APOE* *ε4*, apolipoprotein E allele 4. a: The multivariate analysis adjusted potential confounders, specifically: age, gender, educational level, duration of epilepsy, seizure type, hypertension, diabetes, and dyslipidemia

Compared to participants with *APOE ε4* (-) and low seizure frequency, those with *APOE ε4* (-) and high seizure frequency had an increased risk of CI (OR = 3.01, 95%CI 1.24, 7.29, *P* = 0.015). Participants with *APOE ε4 *(+) and high seizure frequency had an even higher risk of CI (OR = 7.95, 95% CI [1.88, 33.67], *P* = 0.005). After adjusting for age, gender, educational level, duration of epilepsy, seizure type, hypertension, diabetes, and dyslipidemia, those with *APOE ε4* (-) and high seizure frequency showed a threefold risk of CI (OR = 3.34, 95% CI [0.99, 11.25], *P* = 0.051), while those with *APOE ε4* (+) and high frequency demonstrated the highest risk of CI (OR = 10.53, 95% CI [1.75, 63.47], *P* = 0.010) (Fig. 2 and Table 2).

**Table 2:** Multivariate analysis of cognitive function in older people with epilepsy

	β	SE	*P*-value	OR (95%CI)
Age	0.12	0.05	0.007	1.13 (1.03, 1.23)
Male	0.30	0.54	0.581	1.35 (0.46, 3.93)
Education year	-0.39	0.11	0.000	0.68 (0.55, 0.84)
Duration of epilepsy	0.01	0.02	0.687	1.01 (0.97, 1.04)
Seizure type	0.65	1.74	0.709	1.92 (0.06, 58.37)
Hypertension	0.79	0.68	0.246	2.21 (0.58, 8.45)
Diabetes	0.63	1.22	0.607	1.87 (0.17, 20.25)
Dyslipidemia	-0.58	0.58	0.316	0.56 (0.18, 1.74)
APOEε4 (-) and low seizure frequency			Ref	
APOEε4 (+) and low seizure frequency	0.77	0.96	0.421	2.17 (0.33, 14.20)
APOEε4 (-) and high seizure frequency	1.21	0.62	0.051	3.34 (0.99, 11.25)
APOEε4 (+) and high seizure frequency	2.36	0.92	0.010	10.53 (1.75, 63.47)

*Abbreviations*: *APOE ε4* Apolipoprotein E allele 4

## Discussion

In this study, we found CI participants were older, had a lower level of education, and had a higher seizure frequency than UC group. Notably, poorer cognitive performance was observed in global cognition and each specific cognitive domain. In addition, high seizure frequency and more *APOE ε4* allele were identified as risk factors associated with cognitive function. Specifically, participants with *APOE ε4* (+) and high seizure frequency had the highest risk of CI, which means that the *APOE ε4* status may act synergistically with high seizure frequency to exacerbate cognitive decline in older adults with epilepsy.

Our study was consistent with some previous studies. Sekimoto et al. found a significant negative correlation (*ρ* = -0.46, *P* < 0.01) between recent seizure frequency and delayed recall scores in people with late-onset TLE [16]. Rawle et al. used a birth cohort analysis and found that the cumulative detrimental impact of *APOE ε4* on memory from midlife to early old age displays dose-dependent effects (β = -0.68 for heterozygotes vs β = -1.38 for homozygotes) [17]. Gambardella et al. conducted a neuropsychological and molecular study in 138 patients with mild non-lesional TLE who rarely or never had seizures during follow-up and found that *APOE* *ε4* was associated with memory impairment (OR 4.18, 95% CI [1.66, 10.55]). Furthermore, the interaction was found between disease duration and *APOE ε4*. In individuals carrying *APOE ε4* allele, the notable risk of CI reached 32.29 (OR 32.29, 95% CI [5.23, 195.72]) when the disease duration longer than 25.5 years [18]. A longitudinal study showed that older epilepsy patients with *APOE ε4* allele were associated with 1.51 (95% CI [-2.26, -0.75], *P* < 0.001) points faster cognitive decline than expected if epilepsy and *APOE* *ε4* acted independently [19]. However, Doherty et al. compared the executive function of TLE with healthy people and found that APOE ε4 status alone was not a significant predictor of executive function in TLE patients [20].

These studies highlight the multi-factorial nature of CI in epilepsy, implicating both genetic predisposition via the *APOE ε4* allele and clinical characteristics such as seizure frequency and epilepsy duration as critical contributors to cognition. Due to limitations such as cohort size, previous studies have primarily focused on cognitive profiles between individuals with epilepsy and healthy controls [21]rather than exploring the unique impacts of epilepsy-related factors on cognition. To effectively identify cognitive impairment at its earliest stages within the epilepsy population, greater attention should be given to the distinctive effects of epilepsy itself on cognitive function. Moreover, many studies tended to describe clinical characteristics of epilepsy separately, neglecting examining interactions among multiple variables. Our study, however, conducted detailed comparison of epilepsy characteristics, comprehensive evaluation of cognitive function, and multivariate statistical models adjusting for potential confounders. Furthermore, we demonstrated the synergistic effect of *APOE ε4* allele and seizure frequency on cognitive function in older people with epilepsy.

The relationship between the *APOE* *ε4* allele and seizure frequency is based on a myriad of complex mechanisms, encompassing neuronal function, Aβ peptide generation, and the pathophysiology of epilepsy [5]. Aboud et al. used superior temporal lobes of 92 participants and found smaller neurons and more markers of stress in *APOE ε4/ε4* group. These influences may contribute to the increase in frequency and intensity of seizures [5, 22]. Seizure activity may sustain the dysfunction of regional neurovascular unit (NVU), which orchestrates the coupling of neural activity with cerebral blood flow and regulates the integrity of the blood–brain barrier (BBB) to safeguard the structural and functional coherence of the central nervous system [23, 24]. Disruption of NVU integrity leads to alterations in BBB structure or permeability, culminating in dysfunctions that have been implicated in cognitive impairment [25–27]. Concurrently, with advancing age, the decline in neuronal plasticity impedes the ability to compensate the underlying pathological disruptions engendered by seizures, accelerating cerebral aging and cognitive decline [24]. The cumulative effects of recurrent seizures, coupled with interictal epileptiform discharges, exacerbate cognitive deterioration [4, 28, 29].

There were several limitations in this study. First, this study was designed as a cross-sectional study and cannot prove a causal relationship between APOE and seizure frequency synergies and cognitive impairment in older people with epilepsy. The number of patients included in this study mainly depended on outpatient visits, and strict sample size calculation was not carried out. It is necessary to further expand the sample size of the study to enhance the statistical power. Second, the seizure frequency in the past year may not well-represent the long-term situation. Third, previous studies have shown that ASMs may impact cognitive function of people with epilepsy [4, 30]. In our study, about 95% of participants were on ASM treatment. However, the specific ASM types and dosages could not be accurately recalled by the older people with epilepsy. Therefore we could not analysis the impact of ASMs on cognitive function. Forth, the lack of neuroimaging restricts our capacity to comprehensively assess the impact of structural abnormalities and vascular factors on the pathophysiology of epilepsy and cognitive decline. Fifth, defining high seizure frequency as > 3 seizures/year was overly broad and fails to account for the variability within this spectrum. However, due to our limited sample size, we only could make two categories. Future studies should use more granular subgroup definitions such as < 2 seizures/year, 3–4 seizures/year, 1 seizure/month, and > 1 seizure/month. Sixth, overall the sample size of *APOE ε4* carriers was small, and the *APOE ε4/ε4* genotype was limited to three participants. Such small sample size adversely impacts the conclusion of the impact of *APOE ε4* carriers on the neurocognitive assessment. Seventh, due to the limited sample size, our logistic regression analysis using a single categorical variable (four groups with *APOE ε4* and seizure frequency combination) on cognitive impairment may reduce the statistical power of our findings. Future studies with larger cohorts are warranted to confirm these results and, if appropriate, to explore pairwise inter-group comparisons using multiple comparison corrections such as False Discovery Rate (FDR) or Bonferroni, while accounting for relevant confounders. Longitudinal cohorts with larger sample size may reduce bias and enhance statistics power.

## Conclusions

Our study demonstrated the synergistic effect of *APOE ε4* allele and high seizure frequency on worse cognitive function in older people with epilepsy. Our findings may help clinicians to identify individuals with high risk of cognitive impairment and adjust the therapeutic approaches in managing older people with epilepsy.

## Data Availability

The data that support the findings of this study are available from the corresponding author upon reasonable request.

## Abbreviations

ASM: Antiseizure medication
AD: Alzheimer’s disease
PD: Parkinson’s disease
APOE ε4: Apolipoprotein E ε4
MMSE: Mini-Mental Status Examination
UC: Unimpaired cognition
CI: Cognitive impairment
M: Mean
SD: Standard deviation
IQR: Interquartile range
NVU: Neurovascular unit
BBB: Blood–brain barrier
